# Applications of Lignocellulosic Fibers and Lignin in Bioplastics: A Review

**DOI:** 10.3390/polym11050751

**Published:** 2019-04-28

**Authors:** Jianlei Yang, Yern Chee Ching, Cheng Hock Chuah

**Affiliations:** 1 Department of Chemical Engineering, Faculty of Engineering, University of Malaya, Kuala Lumpur 50603, Malaysia; yangjl@gmail.com; 2 Department of Chemistry, Faculty of Science, University of Malaya, Kuala Lumpur 50603, Malaysia; chuah@um.edu.my

**Keywords:** lignocellulosic fibers, lignin, bioplastics, compatibility, hydrophilic

## Abstract

Lignocellulosic fibers and lignin are two of the most important natural bioresources in the world. They show tremendous potential to decrease energy utilization/pollution and improve biodegradability by replacing synthetic fibers in bioplastics. The compatibility between the fiber-matrix plays an important part in the properties of the bioplastics. The improvement of lignocellulosic fiber properties by most surface treatments generally removes lignin. Due to the environmental pollution and high cost of cellulose modification, focus has been directed toward the use of lignocellulosic fibers in bioplastics. In addition, lignin-reinforced bioplastics are fabricated with varying success. These applications confirm there is no need to remove lignin from lignocellulosic fibers when preparing the bioplastics from a technical point of view. In this review, characterizations of lignocellulosic fibers and lignin related to their applications in bioplastics are covered. Then, we generalize the developments and problems of lignin-reinforced bioplastics and modification of lignin to improve the interaction of lignin-matrix. As for lignocellulosic fiber-reinforced bioplastics, we place importance on the low compatibility of the lignocellulosic fiber–matrix. The applications of lignin-containing cellulose and lignocellulosic fibers without delignification in the bioplastics are reviewed. A comparison between lignocellulosic fibers and lignin in the bioplastics is given.

## 1. Introduction

Plastics are widely used in human life. Every year huge amounts of plastics are produced and used in various industrial sectors [1,2,3]. The plastics produced from petroleum are not biodegradable and consequently induce serious environmental issues [4,5,6]. Moreover, the reduction and considerable cost of fossil fuels require alternative and sustainable resources for our future. Therefore, there is extensive research effort on developing biodegradable plastics or bioplastics from sustainable resources for different applications [3,7,8]. Starch, protein, corn-derived poly-lactic acid (PLA), and microorganism-derived poly-hydroxybutyrate (PHB) are the most influential and biodegradable biopolymers in the bioplastic market [9,10,11].

The application of bioplastics has been limited because of their low mechanical strength [12,13]. Numerous studies have demonstrated that synthetic fibers, such as glass and carbon fibers, are commonly used as reinforcements in bioplastics due to their strong mechanical properties. However, the synthetic fibers also cause serious problems to the environment owing to their non-biodegradable characteristic in the environment [14,15,16]. Recently, a wide range of attractive and alternative materials that can replace synthetic fibers such as lignocellulosic fibers are increasingly being utilized as environmentally friendly materials which can reduce the widespread dependence on fossil fuels and enhance the environment and economy simultaneously [16,17,18]. In comparison to synthetic fibers, lignocellulosic fibers are biodegradable, renewable and widely available; moreover, they have low density, competitive specific mechanical properties and a relatively low cost [6,19]. Lignocellulosic fibers such as sugar cane bagasse [20], wheat straw [19], rice straw [1], forest wood [21], flax [22], hemp [12], kenaf etc. [23] have been widely used as reinforcements in bioplastics. Several reviews of lignocellulosic fibers and biopolymers in bioplastics have already been conducted [4].

Along with a number of benefits as reinforcements, there are some drawbacks associated with these lignocellulosic fibers. A significant number of studies focus on improving the compatibility between the lignocellulosic fibers and other biopolymers [9,24]. The hydrophilicity and strong crosslinking of lignocellulosic fibers prevent the compatibility with biopolymer matrices, leading to poor interfacial adhesion and mechanical properties [25,26]. These bioplastics possess limited dimensional stability when exposed to moisture [25]. Therefore, surface modifications are widely employed to enhance the performance of lignocellulosic fibers and to promote better adhesion between the natural reinforcement and the polymeric matrix [27]. Today, most of the surface modifications are based on cellulose by removing the complicated lignin. As a consequence, lignin commonly changes into a waste in the residuals, while cellulose is used in the attempt to make bioplastics [9,28]. Although the surface treatment can attain better performance, but removing lignin requires considerable energy input and produces much wastewater [16]. From a technical point of view, the process removing lignin is demanding technically and profitable impractically [11].

Recently, lignin-reinforced bioplastics have gained the attention of researchers around the world. Lignin is one of the three main ingredients in natural lignocellulosic materials. Lignin is second in plant biomass abundance after cellulose and the most plentiful natural aromatic resource [29,30]. It has been estimated that about 70 million tons of lignin from the paper-making industry are available per year [31,32]. Many studies are being conducted to use lignin in biocomposites including bioplastics because of its wide availability, good mechanical properties, biodegradability, besides the various modifications based on its chemical structure [4,33,34,35]. Lignin has been incorporated into many biopolymers, such as starch, protein, cellulose, PLA and PHB, to form bioplastics [4,31].

The first section of the review focuses on specific characterizations of lignocellulosic fibers and lignin. The second section summarizes the recent advances and issues of lignin in the development of bioplastics. The third section reviews some challenges inherent in the application of lignocellulosic fiber in bioplastics and the means attempted by researchers to modify their properties. Finally, we give an outlook on their future applications in bioplastics.

## 2. Structure and Properties of Lignocellulosic Fibers and Lignin

Lignocellulosic fibers are the largest source of renewable bioresources in the world. Generally, lignocellulosic materials are composed of 35–55 wt % cellulose, 10–25 wt % lignin and 20–40 wt % hemicellulose in addition to extracts (e.g., pectin, resins, waxes, etc.), ash and minerals [36,37]. The compositions of cellulose, hemicellulose and lignin in a few lignocellulosic biomasses are shown in Table 1.

**Figure 1:**
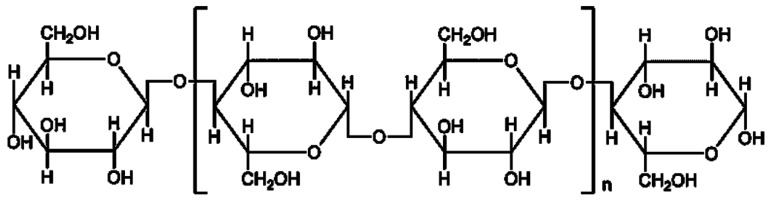
Cellulose structure (Reprinted with permission from [45]. Copyright 2014, Elsevier).

Cellulose is a linear biopolymer made up of 7000–15,000 d-glucose monomers which are connected via β-1,4-glycosidic linkages (Figure 1) [42]. Cellulose chains are bonded by van der Waals forces and hydrogen bonds in the microfibrils. In the different cell walls, the arrangement of microfibrils is also different. Microfibrils are combined to form the cellulose fiber [38]. Crystalline cellulose appears in the form of crystallinity [43]. Degree of polymerization of cellulose is between 1510 and 5500, which strengthens its crystallinity. Moreover, amorphous cellulose is non-organized, which takes up a small proportion. Crystalline cellulose is more resistant to degradation of enzyme compared to amorphous cellulose [42,44].

**Figure 2:**
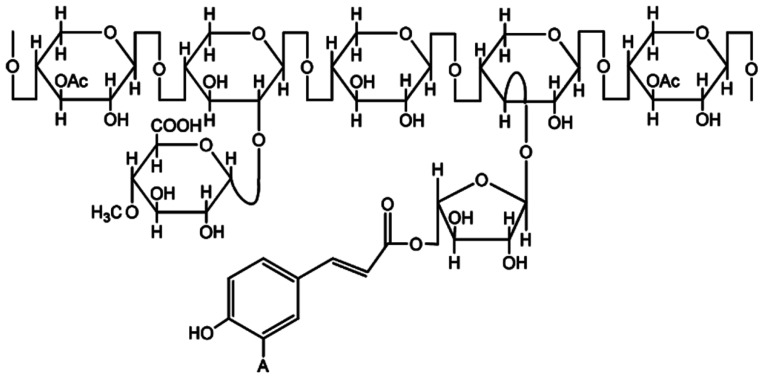
Representative hemicellulose structure (Reprinted with permission from [45]. Copyright 2014, Elsevier).

Hemicellulose is an amorphous and heterogeneously branched polymer of pentoses and hexoses, mainly d-galactose, d-xylose, d-mannose, l-arabinose, d-glucose, with 500–3000 sugar monomers [38]. β-1,4- and occasionally β-1,3-glycosidic bonds are the main bonds between sugars. The representative structure of hemicellulose is shown in Figure 2. Hemicellulose has a lower molecular weight in contrast to cellulose [42]. Hemicellulose has a degree of polymerization between 50 and 200, which makes it amorphous and easily hydrolysable. When hemicellulose is co-crystallized with cellulose it does not aggregate [40,44].

**Figure 3:**
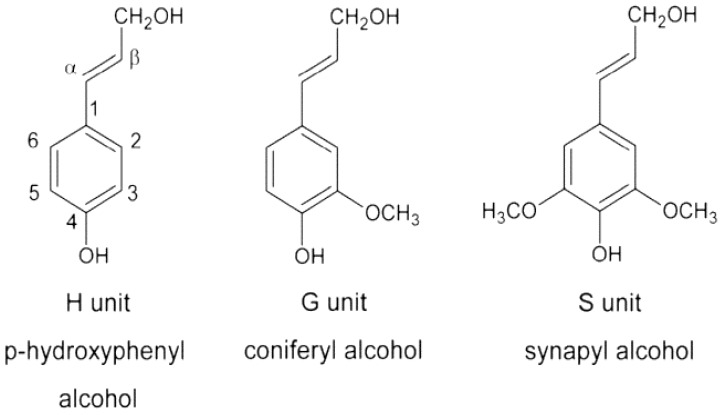
Three lignin monomers (Reprinted with permission from [53]. Copyright 2014 Elsevier).

Lignin differs from cellulose and hemicellulose as it contains aromatic rings rather than long molecular chains. Depending on the different plant and extraction process, lignin is characterized by diverse chemical structures [29]. The most essential differences are found in the monomer composition, linkage type and functional group in the lignin fragments [46]. Lignin is a polyphenolic macromolecule, which consists of three phenylpropane monomeric units, coniferyl alcohol(G), p-hydroxyphenyl alcohol(H), and sinapyl alcohol(S) (Figure 3). The coniferyl alcohol structure dominates in softwoods. Lignin in hardwood commonly contains both sinapyl alcohol and coniferyl alcohol structures with sinapyl alcohol dominant, while p-hydroxyphenyl alcohol structure predominates in lignin found in grasses [30,47]. Different types of carbon-oxygen (aryl-ether) and carbon-carbon bonds are formed in different subunits of lignin. The most frequent bonds are the carbon-oxygen links between β-end of the propenyl group (β-O-4) and p-hydroxy moiety [44,48]. Different percentages of chemical groups in the lignin molecule structure such as methoxyl, hydroxyl, carbonyl, carboxyl et al. impart polarity to the lignin macromolecule. The dominant chemical groups are hydroxyl groups which are aliphatic or phenolic [46,49]. As the most recalcitrant component in lignocellulosic fibers, lignin is extremely resistant to enzymes and chemical impacts [50]. Lignin does not dissolve in hot water, acids, and other solvents except alkalis [38,51,52].

Because of its complicated structure, the molecular weight is an essential parameter of lignin [50,54]. Molecular weight of lignin is from 1000 to 20,000 g/mol. Lignin is widely fragmented in the extraction process and contains types of subunits which repeat randomly. Therefore, its degree of polymerization is hard to analyze [4,55]. Besides the molecular weight, another imperative parameter that affects the properties of lignin is the glass transition temperature (*T*_g_) [4] (Table 2). In most of the pulping process, *T*_g_ of lignin ranges from 100–170 °C. It is higher than the *T*_g_ of most synthetic materials [56]. Yoshida et al. found that the degree of association by hydrogen bonding from the phenolic hydroxyl groups primarily contributed to the high *T*_g_ of lignin [57]. The high *T*_g_ is also attributed to the chemical structure of lignin, particularly the aromatic ring of the main chain [32]. *T*_g_ is considered as the most suitable parameter to assess polymer miscibility [58]. Complete compatibility in the polymeric blend can be implied by a single *T*_g_ which is an average *T*_g_ of each component. Two *T*_g_s or above indicate low compatibility [59].

Broadly speaking, lignocellulosic fibers are made up of cellulose fibers reinforced by a matrix of hemicellulose and either lignin or pectin in one or more layers, with the volume fraction and orientation of the cellulose fibers varying in each layer [42]. The cellulose molecules are hydrogen-bonded to hemicellulose. The cellulose-hemicellulose network is considered as the primary structural component of the fiber cell [36]. Then, lignin covalently linked to hemicellulose strengthens the structure of cellulose-hemicellulose [62,63]. The hydrophobic lignin functions as a coupling agent filling in the voids that exist in the cellulose-hemicellulose network. Lignin improves the stiffness of the cellulose-hemicellulose network and protects cellulose fibers against biological attack and environmental pressure [30,64,65]. Eight types of bonds are present in lignin-carbohydrate including benzyl ester, benzyl ether, hemiacetal or acetal linkages, glycosidic or phenyl glycosidic, and diferulate or ferulate esters which link with lignin at the 4-O and 4-OH positions [66]. Different proportions and types of polysaccharides and lignin form lignin-carbohydrate complexes (LCC) showing a various compositions and structures. LCC render cell walls recalcitrance for biorefining [67,68,69].

## 3. Application of Lignin in Bioplastics

It has been estimated that around 70 million tons of lignin in the paper-making industry are available per year [31,32]. However, only 2% of it is processed and utilized as lignin, the rest is added into fuels [53,64]. Commercially available lignin obtained from industrial delignification methods is called technical lignin (Table 2). One main source of technical lignin is provided by the paper-making industry with two major lignins: Kraft lignin and lignosulfonates [4]. Kraft lignin (KL) constitutes about 85% of total world lignin production, but lignosulfonates are the most important commercially available lignin whose yield is approximately 1 Mt yr1 [53,70]. The category comprises almost the total market of commercially available lignin [71]. The second main lignin source is mainly related to the soda pulping process and Organosolv pulping processes. The category is closer to the native structure of lignin because it is sulfur-free. Today, the soda process is utilized primarily to extract cellulose from low lignin content of non-wood crops such as wheat straw, bagasse, hemp, kenaf or sisal. However, it is generally hard to recover soda lignin through centrifugation or filtration due to its high content of carboxylic groups arising from aliphatic hydroxy group oxidation. This causes lignin to be highly hydrophilic and renders lignin a potential dispersant. Organosolv lignin is a less modified lignin. The homogeneity of Organosolv lignin is higher than that of lignosulphonates or KL. Organosolv lignin has the lowest molecular weights among the technical lignin and can be dissolved in certain solvents, which facilitates their further processing [72]. In addition, there is ionic liquid lignin, steam explosion lignin, enzyme lignin etc. [73]. Since lignins originate from different sources and separation processes, they differ from each other in terms of chemical structure, composition, and reactivity to a significant extent. As a result, each lignin type needs to be considered individually [72]. The first proportion of this section mainly covers the direct use of lignin with starch, protein, cellulose, PLA and PHB in bioplastics. In the second part, the field of modified lignin reinforced bioplastics is addressed.

### 3.1. Lignin as Reinforcements in Bioplastics

Blending lignin with other biopolymer materials has been attractive because of its wide availability, good mechanical properties, biodegradability, along with the diversity of potential modifications due to its chemical structure [35]. Many studies have focused on the incorporation of lignin into natural biopolymers, such as starch, protein, cellulose, PLA, and PHB, to form bioplastics [4,11,30,34]. The addition of lignin as reinforcements typically reduces cost and water uptake and improves the strength [74]. In addition, lignin plays an important part in antioxidant properties as a stabilizer because the phenolic hydroxyl groups can scavenge free radicals [46,75]. Plasticizers can interact with the polymer by replacing polymer interactions [60]. This phenomenon improves the flexibility, mobility and workability of polymer by reducing intermolecular forces, *T*_g_ and the processing temperature of the blends [76]. Plasticization has been observed when lignin is introduced into starch, protein and polycaprolactone [29,53,64,77,78].

The lignin molecule is relatively polar due to its numerous hydroxyl groups and generally presents good compatibility with polar polymer matrix. Therefore, lignin is immiscible with most polymers [79,80]. This is often related to strong intermolecular interactions of lignin which result in poorer interactions between the polymer and lignin. The resulting bioplastics show poor adhesion and dimensional stability [25]. Various chemical groups containing oxygen, primarily hydroxyl groups, lead to the high degree of interaction in lignin molecules [60]. Moreover, lignin tends to aggregate in biopolymers owing to the p-p stacking of hydrogen bonding, its aromatic rings and van der Waals attractions between the polymer chains, which can impair the properties of the resulting composites. Consequently, the properties of the composites are determined by competitive interactions. Obviously, the interactions developed in the blends such as hydrogen bridges are not sufficient to form complete compatibility. The deformability of the blends might be compensated for by using the compatibilizers and coupling agents in the bioplastics [74,81]. In addition, plasticization is possible to reduce the *T*_g_ and improve the affinity [60].

#### 3.1.1. Starch-Lignin Bioplastics

Starch is considered as the most widely used biopolymer as a replacement for conventional plastics due to advantages such as high abundance, low cost, renewability and its ability to be degraded without the formation of toxic residues [82]. However, its apparent drawback is low water resistance and mechanical strength [83]. Reinforcements, either microparticle or nanoparticle, have been incorporated into starch in order to overcome these inherent shortcomings [84,85]. In general, the properties of starch-lignin blends are mainly determined by the compatibility between their components. The lignin is dispersed into starch through the interactions of hydrogen bond. Films based on starch and lignin bioplastics reinforced by graphene oxide were fabricated by casting and solvent evaporation method successfully. Fourier transform infrared (FT-IR) spectroscopy indicated that a homogenous biocompatible blend matrix was formed through the presence of a hydrogen bond. After the introduction of graphene oxide, additional interactions were found between the hydroxyl groups of lignin and starch and the groups of graphene oxide containing oxygen, forming an interacted network. The properties of the resulting bio-nanocomposites such as water uptake, water swelling, hydrolytic degradation, water vapor permeability, mechanical and thermal properties were enhanced significantly due to these interactions [86].

Blending starch with hydrophobic polymers typically increases the water resistance, mechanical strength and thermal stability of starch. Lignin is an obvious candidate for blending with starch because of its high miscibility [84,85]. Films containing wheat starch and KL were produced by extrusion and hot molding. Stress-strain tests were used to evaluate the influence of lignin content on the mechanical properties of films at two ambient humidity. Stress and elongation at break increased slightly with 20% lignin and 58% relative humidity. Compared to the control group, a significant reduction of film resistance to elongation was observed at 71% relative humidity and 30% lignin. Water sorption isotherms and dissolution analysis revealed that lignin reduced the total water uptake of the films [87]. Souza de Miranda et al. (2015) evaluated the morphological and mechanical properties of thermoplastic corn and cassava starch films, using lignin and glycerol as reinforcements and plasticizers. The results indicated that the addition of lignin significantly improved the elastic modulus and the maximum stress by about 4200% and 840%, respectively, compared to the sample without lignin. Moreover, lignin caused the rougher surface of the film, modified some structural properties, and improved thermal properties [88]. Stevens et al. (2010) have prepared novel starch-KL foams. X-ray diffraction (XRD) and differential scanning calorimetry (DSC) together revealed that there were significant starch–lignin interactions that stabilized residual structure in the starch. Lignin decreased water absorption of the foams. Starch–lignin foams prepared by the present method had approximately the same flexural strength as foamed polystyrene [13].

To enhance the properties of starch–lignin bioplastics, starch is modified and then blended with lignin to make bioplastics. Starch-lignin biocomposites were fabricated with modified starch microparticles originating from cross-linked starch containing adipic acid and glycerol. The thermal stability and the water resistance of composites were increased via the introduction of lignin. Starch–lignin composite materials presented a lower elongation at break, but higher tensile strength compared with those without lignin [89]. The lignin with 5%, 10%, and 15% content was incorporated to improve the water resistance of the urea crosslinked starch films. To investigate the water uptake behavior, the produced films were placed in distilled water at three temperatures, 25, 35 and 45 °C. Lower water uptake equilibrium confirmed that the addition of lignin decreased water uptake effectively due to the hydrophobicity of lignin [90].

The addition level and sources of lignin greatly influence the performance of starch bioplastics. The composition of lignin will also determine the final properties of the bioplastics at high lignin percentages. Many reports found the 10% of lignin content can produce the optimal performance of bioplastics. A biodegradable starch/lignin film was fabricated by corn starch and sodium lignosulfonates. The effect of lignosulfonates on hydrophobicity and the mechanical properties of films was identified. The ultimate stress was reduced, and the hydrophobicity of films was improved with the lignosulfonates to starch ratio increasing from 1:9 to 9:9. The results from a scanning electron microscope (SEM), FTIR, and XRD revealed that there was good compatibility among lignosulfonates, starch, and sorbitol [91]. Vengal and Srikumar (2005) reported the results of water absorption and tensile tests of different starch-lignin polymer films. The study adopted solution blending followed by curing for the preparation of the films. The optimal proportion of starch to lignin was 90:10, as it showed high Young’s modulus and tensile strength. The water uptake tests showed that the system was highly hydrophilic. The material was suitable for internal use and biomedical purposes [5]. Wheat starch and crude lignosulfonates with glycerol as plasticizer were used to prepare films by hot molding and casting. Incorporating 10% lignosulfonates modified the tensile resistance of the films, independently of the composition of the lignosulfontes; When the addition of lignin was above 10%, sodium and calcium lignosulfonate-based films showed different mechanical performance. A single *T*_g_ varying from 16 to 40 °C, which resulted from different lignin contents, was revealed by dynamic mechanical thermal analysis [92]. Starch-lignin films were produced with lignin content from 1.2 to 2.4 wt %. As the lignin increased in the biofilms, thermal and mechanical properties of the biofilms did not attain the maximum [83]. Bhat et al. (2013) studied the barrier and mechanical performance of starch-lignin based films. Food packaging films were produced from sago palm starch and lignin separated from oil palm black liquor (1–5% *v*/*w*) by casting method. The results indicated that lignin improved thermo-mechanical, barrier and water resistance properties of the films [85]. The inconsistence might be due to the different compositions or sources of lignin and the different preparation methods of bioplastics.

The plasticization of lignin on starch is reported. Baumberger et al. (1998b) investigated that proportions of KL in starch bioplastics could lead to great changes of mechanical strength: high molecular weight led to very brittle materials whereas low molecular ones functioned as a plasticizer. The mechanical properties of the bioplastics were significantly affected by the low molecular weight lignin in small quantities due to the plasticizing effect [77]. The literature on plasticization of lignin in the starch-lignin bioplastics is scarce. There are several reports about the plasticization of lignin on other biopolymers such as protein and PLA.

#### 3.1.2. Protein-Lignin Bioplastics

As a natural polymer, proteins have long been used to produce bioplastics owing to their abundant, biodegradable, renewable, and environmentally friendly features [93,94]. However, the performance of bioplastics derived from protein such as water absorption, thermal stability and mechanical strength is relatively lower compared to other common plastics [95,96]. Lignin has been evaluated to enhance the strength and water resistance as a filler in protein-based bioplastics. Strong hydrogen bonding between the amino acids of proteins and the functional groups of lignin generates a reconstruction of intra- and intermolecular interactions in secondary structures of β-turn, β-sheet, and R-helix. This restructuring produces an extensive conformational modification of protein which, conversely, causes a great increase on the physical properties of the biocomposites [97].

Lignin can improve the properties of protein-lignin bioplastics in high percentages, which may hold promising application as a replacement of protein. Chantapet et al. (2012) evaluated the function of KL on the properties of new resulting bioplastics and on the extrusion process. Wheat gluten (WG) was extruded with KL in a corotating twin screw extruder with circular die. The processability of WG in extrusion was enhanced by the KL with 10–50 wt % contents: both residence time and die pressure for all mentioned screw speeds and feed rates were decreased. The introduction of 10–30 wt % KL reduced elongation at break and water absorption of KL/WG materials, and enhanced Young’s modulus and tensile strength [98]. Fish protein-reinforced bioplastics were explored to investigate the effect of KL on protein rheological and functional properties and aggregation. Fish protein powder was blended with 0–70% KL and 30% glycerol. Compression molding was used to develop the bioplastics. Solubility of protein increased in the presence of KL in sodium dodecyl sulfate buffer, which indicated that the molecular weight of the protein decreased. Mechanical properties increased with the introduction of KL, while KL addition caused a decrease of water absorption and storage modulus of the bioplastics. A capillary rheometer revealed that the viscosity also decreased at experimental temperature [94]. A variety of bioplastics from lignosulfonates and soy protein isolate with a weight ratio of 4:6 to 10:0 were fabricated through compression molding. The outcomes reported that the content of lignosulfonate from 30 to 40 parts could significantly improve Young’s modulus, tensile strength and elongation at break. Water uptake analysis of the bioplastics suggested that the effect of water on the swelling and the damage of the bioplastics was restricted by the intense interaction between soy protein isolate and lignosulfonates. The improved properties were attributed mainly to the formation of crosslinked structures between lignosulfonates and soy protein and the occurrence of the beneficial microphase separation [93].

Lignin can act as a stabilizer in protein-lignin bioplastics due to the radical scavenger activity of lignin. KL was mixed with WG and incorporated into materials by thermal molding. The results proposed a radical scavenger activity of KL to the thiyl radicals was observed during material mixing. Size exclusion high-performance liquid chromatography (HPLC) confirmed the good connection of WG and KL [99]. Different concentrations of alkali lignin and lignosulfonates were introduced to enzymatically modified soy protein in order to improve physical and functional properties of the bioplastics. Compared to commercial butylated hydroxytoluene, alkaline lignin and lignosulfonates indicated typical radical scavenger activity, which presented possible potential application in active packaging. Due to the color of lignin, the blends with alkaline lignin revealed strong ability of ultraviolet (UV)-blocking. Compared to the control films without lignin, thermal stability and mechanical strength of the films including lignin were enhanced significantly [100].

Contradictory results on mechanical strength, water uptake and *T*_g_ are reported by several studies. The phenomenon is attributed to the type of lignin. Ammonium lignosulfonates and KL have been combined to make WG-reinforced bioplastics. The addition of KL resulted in increased Young’s modulus and mechanical strength, while the elongation at break remained constant. Water vapor sensitivity was reduced and *T*_g_ became higher as KL was introduced. By contrast, elongation at break became higher, and water sensitivity and *T*_g_ were unchanged in the presence of lignosulfonates. These effects were usually observed from plasticizers. It suggested that commonly used glycerol in WG could be replaced by lignosulfonates. The addition of lignosulfonates did not induce a decrease in the glass transition temperature or an increase in moisture absorption, which was different from the incorporation of glycerol [29]. Kunanopparat et al. (2012) surveyed the physical properties and processability of WG-KL bioplastics. The KL in bioplastics contributed to a dramatic decrease of the rubbery storage modulus but an increase of the *T*_g_. Important interaction between WG and KL was demonstrated by the increase in *T*_g_. Compared to the original WG bioplastics, the prepared biocomposites exhibited lower water sensitivity along with higher tensile strength effectively [101].

In addition, colloidal lignin nanoparticles and lignin particles were coated by proteins and the composites were characterized. Atomic force microscopy and transmission electron examined the morphology of individual lignin particles coated with protein. The enhanced adsorption ability to specified amino acid residues such as proline and serine was evidenced by FT-IR and capillary electrophoresis. As the surface chemistry of colloidal lignin particles was tailored by the protein corona, the colloidal lignin particles presented significant applications in bioplastics and biomedicine [96].

#### 3.1.3. Cellulose-Lignin Bioplastics

The compatibility between cellulose and other hydrophobic biopolymers could be remarkably enhanced by the amphiphilic lignin with polar phenolic OH and non-polar hydrocarbon groups [34]. Therefore, lignin is a potential component for bio-compatibilizer [4,64]. The addition of lignin could improve the compatibility between cellulose and matrix as shown by scanning electron microscope (SEM) investigations. The impact properties are decreased, while Young’s modulus and tensile strength could be improved significantly [31].

Cellulose and lignin play different parts in cellulose–lignin bioplastics. In general, cellulose reinforces mechanical strength of the composites, while lignin reduces water uptake, improves thermal stability of the polymer matrix and assures the good dispersion of cellulose in biopolymers [102,103]. They can generate mutual effects on the bioplastics [104]. A number of bio-based composites based on starch, lignin and cellulose were fabricated from an ionic liquid, 1-allyl-3-methylimidazolium chloride and coagulated in a system without solvent. The study found that the mechanical strength of the biocomposites was evidently depending on the contents of lignin, starch and cellulose, resulting from the mutual supplement among different components. High gas barrier ability and great thermal stability were clearly observed in the biocomposites [105]. Holo-cellulose and acid insoluble lignin of Pecan nutshell fiber were utilized as reinforcements of PLA-based bioplastics. Flexural tests demonstrated that modulus of holo-cellulose biocomposites increased by 25% in comparison with the pure biopolymer. Conversely, high ductility and improved elongation at break were provided by the acid insoluble lignin in the PLA bioplastics. Plain PLA showed higher resilience than all the other biocomposites owing to the low compatibility between lignin and PLA [106]. Moreover, isolated lignin and holo-cellulose components were blended with PHB. PHB melt crystallization was facilitated by holo-cellulose in the cooling period as evidenced by DSC investigation, while this phenomenon was not influenced by lignin. The rheological investigation of PHB and PHB-based blends indicated polymer viscoelastic properties improved remarkably. The high melt viscosity of PHB/lignin sample hampered chain mobility. The research provided a new point of view regarding the impact of cellulose and lignin on PHB [107].

The plasticization properties and antibacterial activity of lignin are also observed in cellulose-lignin bioplastics. In addition, the addition content of nanoparticles is generally restricted below 3% due to strong inter-molecular hydrogen bonds. Miranda et al. (2015) evaluated the mechanical properties of corn starch bioplastics with lignin serving as a plasticizer and cellulose nanocrystals acting as the reinforcing filler. The outcomes indicated that the incorporation of 1% cellulose nanocrystals and lignin significantly improved the modulus of elasticity and the maximum stress of about 1478% and 256%, respectively, compared to the group without lignin and cellulose [108]. Ternary PLA-based bioplastics, with dispersed lignin nanoparticles and cellulose nanocrystals were produced by melt extrusion at contents of 1 and 3 wt %. The ternary systems presented higher modulus and strength values than those of binary PLA bioplastics and pure PLA. Furthermore, cellulose nanocrystals and lignin nanoparticles have shown antibacterial activity by reducing the bacterial pathogen multiplication because of the polyphenolic structure [46]. In addition, cellulose-lignin nanocrystals can function as outstanding fillers for PLA to develop the bioplastics. To improve the thermo-mechanical and rheological properties of PLA biocomposites, cellulose nanocrystals coated with spray-dried lignin were combined. The results found that the lignin-coated cellulose nanocrystals enhanced their interfacial interaction with the PLA biocomposites and promoted the dispersion of cellulose nanocrystals, leading to a significantly improved thermo-mechanical and rheological properties. Nucleating sites at high density were induced by the super compatibility and dispersion of lignin-cellulose nanocrystals in PLA, generating an increase in PLA crystallinity. Adding 0.5 wt % lignin-cellulose nanocrystals to the PLA biocomposites induced a nearly 60% increase of storage modulus compared to pure PLA. This improvement of mechanical properties was derived from a remarkably improved crystallinity of PLA [109].

#### 3.1.4. Poly-Lactic Acid (PLA)-Lignin Bioplastics

PLA is a biodegradable and crystalline thermoplastic biopolymer presenting good mechanical properties and high recyclability for industrial applications in bioplastics. On account of the high cost of PLA, development of fully bio-based composite with superior properties by introducing lignin into PLA is becoming an important research topic and has attracted great attention in the past few years [35].

Innovative PLA-lignin biocomposites are recommended due to the underlying interaction between the carboxyl groups of PLA and the hydroxyl groups of lignin [35]. The addition of lignin affects the thermal stability and tensile strength of PLA significantly. Wang et al. (2015) reported that the preparation of lignin/PLA-based fibers was attempted by melt spinning, thermal stabilization and carbonization of lignin/PLA fibers. The tensile modulus of the blended carbon fibers was improved by the strong hydrogen bonding interaction between PLA and lignin, confirmed by FT-IR and DSC. However, its tensile strength was decreased primarily attributed to numbers of voids produced in the thermal stabilization and carbonization processes [110]. The role of lignin as fillers in PLA-based polymeric composites was studied. PLA biocomposites with lignin contents from 5 to 15 wt % were fabricated by using injection molding. As the filler content increased, the tensile strength of the biocomposites fell. Compared to pure PLA, PLA-lignin biocomposites started to degrade at a little lower temperatures [111]. The effect of lignin loading on the mechanical and morphology properties of the obtained PLA-lignin biocomposites was identified. The addition of 7 wt % lignin resulted in a decrease in the tensile strength and an improvement of the Young’s modulus. The subsequent increase of lignin levels from 7 to 15 wt % generated a significant improvement of tensile strength [112].

Aggregation of lignin at the nanoscale will also lead to poor properties of PLA-lignin bioplastics. PLA bionanocomposites, which were blended lignin nanoparticles with 0, 1 and 3 wt % contents, were fabricated by melt extrusion and solvent casting. A decreased tendency of tensile strength was observed in the biocomposites, ascribed to inhomogeneous dispersion of lignin nanoparticles; nonetheless, the introduction of lignin nanoparticles affected elongation at break of the biocomposites positively. Chemical, morphological and thermal properties confirmed that the PLA biocomposites’ disintegration was limited by 1 wt % lignin nanoparticle content because of its hydrophobic nature. Rougher surface structure and aggregation led to higher degradation rate When the nanoparticles content rose up to 3 wt % [113]. The poor performance of lignin nanoparticles in melt extrusion is associated with strong inherent hydrogen bonding of lignin. Nanoparticle content in PLA bioplastics is also restricted.

To promote the compatibility with PLA-lignin bioplastics and limit the negative properties of lignin, compatibilizers, such as polyethylene glycol, maleic anhydride, diglyme and phytic acid are generally introduced. Ternary biocomposites containing poly-l-lactic acid, polyethylene glycol and softwood KL have been formed. However, in comparison with the poly-l-lactic acid/lignin system, the biocomposites where poly-l-lactic acid was plasticized with 30 wt % polyethylene glycol and lignin indicated higher deformability. An excellent balance between stiffness and flexibility has been attained in the ternary biocomposites as the content of polyethylene glycol increased [73]. PLA, polyolefin grafting maleic anhydride and cellulolytic enzyme lignin were blended and used to produce the bioplastics by extrusion. The study found that the mechanical properties changed as the contents of components varied in the bioplastics. In contrast with original PLA, the Young’s modulus and tensile strength of the biocomposites decreased, but the impact strength and the elongation at break improved effectively. SEM observations revealed that the cellulolytic enzyme lignin acted as a bridging function between PLA and polyolefin grafting maleic anhydride, promoting their compatibility and resulting in the improvement of toughness and ductility of the blends [114]. Different amounts of diglyme were introduced into PLA biocomposites in order to avoid fiber agglomeration during blending. The tensile strength followed a positive and lineal relationship with fiber contents in the diglyme-containing biocomposites. In addition, the tensile strength of PLA biocomposites reinforced by 30 wt % lignin presented the same amount as polypropylene composites reinforced by 20 wt % glass fibers, which might pave a way to substitute the synthetic composites [115]. Lignin, phytic acid and PLA combinations were used to develop bio-based flame-retardant systems. The composites were prepared by melt blending. Incorporating these two additives has been shown to present an effective way to limit the negative effect of each of them and improve the properties of the produced composites. On the first hand, lignin reduced the composite hygroscopy significantly with the aid of phytic acid. On the second hand, the addition of phytic acid generated better dispersion of lignin into the matrix, which restricted PLA thermal degradation. Some positive effects of the combinations on the mechanical properties have also been confirmed. The elongation at break increased from 3.1% to 12.6% for one formulation [116]. To fabricate PLA bioplastics, PLA, silver nanoparticles and Organosolv lignin were incorporated into polymer blends with lignin serving as a reducing agent. Incorporation of silver nanoparticles and lignin did not change the chemical structure of PLA, as revealed by FT-IR results. The water vapor barrier and mechanical properties of the bioplastics increased after incorporation of silver nanoparticles and lignin. The bioplastics with silver nanoparticles presented potential antimicrobial activity against *Listeria monocytogenes* and *Escherichia coli* [117]. In order to make compatible PLA/lignin blends, triallyl isocyanurate was blended. The formation of PLA-lignin crosslinked structures was confirmed by FT-IR and DSC studies. The compatibility of the resulting blends was further validated by observing the significant improvement in the thermal and mechanical properties, the surface morphology, and *T*_g_ behaviors of PLA/lignin blends [118].

#### 3.1.5. Poly-Hydroxybutyrate (PHB)-Lignin Bioplastics

Polyhydroxyalkanoates are made up of a set of linear and biodegradable aliphatic polyesters with R-(-)-3-hydroxyalkanoate dominant. They provide important energy and carbon storage sources to a variety of bacteria and algae. The most widely used unit in the Polyhydroxyalkanoates family is the PHB [26,119]. PHB is an excellent candidate to prepare bioplastics because it maintains high barrier capacity to carbon dioxide, oxygen and water, and does not dissolve in water, which indicate great advantages over most biopolymers [120].

Compatibility of the biocomposites is related to strong hydrogen bonding interaction present in the carbonyl groups of PHB and the phenol hydroxyl groups of lignin. The present studies indicate that good compatibility is achieved but the tensile strength and thermal stability are reduced. The miscibility and thermal properties of soda lignin and PHB bioplastics were explored. Intermolecular interactions between soda lignin and PHB in bioplastics with 40 wt % lignin content were enhanced remarkably as suggested by the DSC, thermogravimetric analysis (TGA) and SEM results. These results showed good correlation with the *T*_g_ because the blends presented a sole *T*_g_. Two *T*_g_s which depicted immiscibility were obtained at a higher content of lignin in bioplastics [59]. The rheological and thermal properties of lignin and PHB bioplastics were examined and analyzed by DSC, TGA and rheological tests. The lignin reduced the thermal stability of PHB because the activation energy of decomposition decreased from 112 kJ·mol^−1^ of neat PHB to half in PHB/lignin bioplastics apparently. The rheology data showed that lignin developed a single phase with PHB when lignin content was below 30 wt % and acted as a plasticizer (as indicated by scanning electron microscopy and glass transition temperature). The viscosity and elasticity were reduced in comparison to raw PHB. Phase separation took place in 60 wt % lignin content and thus the blend viscosities were increased and the ability to dissipate energy of the blends was decreased [119]. The literature on PHB-lignin bioplastics is scarce. However, as the application of lignin in bioplastics is receiving great attention and the studies of lignin in bioplastics is promising.

From the application of lignin with five biopolymers, we can see that when lignin functions as the reinforcement or plasticizer, opposite effects on mechanical strength and water uptake are induced. Whether lignin will act as the reinforcement or plasticizer is still not clearly studied. The different content and type of lignin incorporated into bioplastics will remarkably influence the properties of the resulting bioplastics. The starch, protein and cellulose reinforced bioplastics with the addition of lignin commonly generate better water resistance, tensile strength and thermal stability, while PLA and PHB with the presence of lignin show lower tensile strength and thermal stability. This phenomenon might be associated with the different structure and properties of biopolymers. Compatibilizers and crosslinkers promote the effect of lignin in these bioplastics by increasing compatibility. The addition content of lignin in these bioplastics can be as high as 10–30% with intended application, which holds potential alternative to substitute the biopolymers. The introduction of lignin nanoparticle is limited within 3% content due to the stronger intermolecular interaction than the interaction between lignin and other biopolymers, especially when the bioplastics are produced by melt extrusion. In order to avoid secondary particles aggregation, incorporation of lignin must use lignin slurry instead of powder in the process of preparation [121].

### 3.2. Functionalization of Hydroxyl Groups of Lignin in Bioplastics

High polydispersity is one of the major problems affecting the applications of lignin. Lignin is commonly treated to enhance its compatibility with biopolymers or to modify its dispersibility in the polymer blends [60]. Based on functional groups and complex structure, modification of lignin to enhance the properties of lignin mainly uses the following approaches: modifying the structure by functionalization of hydroxyl groups, decreasing the molecular weight by depolymerization, and isolating lignin fragments with a specified molecular weight and structure [36,53,122]. Lignin depolymerization is a very promising process that can generate value-added low weight molecules for fuels and basic chemicals or oligomers for further application [68,123]. After chemical extraction, industrial lignin generally has high levels of polydispersity which is from 5 to 10 [54]. Many studies have been conducted to separate fractions of lower polydispersity in recent years, such as selective precipitation, ultrafiltration and solvent fractionation [124]. For example, the impact of diverse lignin derived from a solvent fractionation technique on cellulose-starch bioplastics was reported by Zhao et al. [125]. This research emphasized the lignin diversities on biocomposites properties, indicating that lignin’s applications in bioplastics were determined by their specific features.

Functionalization of hydroxyl groups of lignin is typically used to render it suitable for the intended applications by altering the surface character of lignin [125]. Two types of functionalization of the hydroxyl groups have been carried out. The first method is by connecting the hydroxyl groups of lignin with biopolymer chains, thus improving lignin’s reactivity. The second method involves alkylation or esterification reactions in hydroxyl groups, which facilitates the compatibility of biopolymer matrices with lignin [53]. As a result, the amount of hydroxyl groups will be reduced and hence the water resistance of the blends is greatly improved [64].

To improve the properties of PLA-based bioplastics, lignin is modified and then processed in the bioplastics. The thermal stability, mechanical strength, and antimicrobial properties show different changes depending on the different modification of lignin. Dodecanoyl chloride fatty acid was used to modify lignin obtained from Organosolv process. Different concentrations of esterified lignin (1, 5, 10, 25 and 50%) was incorporated into PLA bioplastics produced by solvent casting. Thermal, water barrier and mechanical properties of resulting films were studied. Esterified lignin still had the phenolic radical scavenging property. The introduction of modified lignin contributed to lower stiffness and greater ductility, which provided plasticity to PLA [126]. Different proportions (0.5%, 1%, 5%, 10% and 20%) of Organosolv lignin and commercial alkali lignin were prepared from almond shells and used to prepare PLA blends by the extrusion method. In order to improve their compatibility with PLA, both lignin was acetylated. The thermal stability of PLA bioplastics was improved by lignin content. Maximum strength decreased as original lignin content increased. However, even at high lignin content, mechanical strength of PLA/acetylated lignin bioplastics still remained unchanged. The elongation at break increased in all composites [127]. PLA was combined with alkylated lignin (contents from 10% to 50%) to synthesize copolymers by ring-opening polymerization. Nanofibrous composites were fabricated by combining these PLA-lignin copolymers with grafted PLA by electrospinning. The outstanding compatibility between the PLA-lignin and grafted PLA was displayed clearly in nanoscale. The mechanical properties of the bionanocomposites were not enhanced by the introduction of PLA-lignin copolymers, which was different from bulk materials. Copolymers or nanofibers with the lignin all presented prominent radical scavenging capacity for more than 72 h as shown by antioxidant assay. Owing to excellent biocompatibility and antioxidant activities, the grafted PLA/PLA-lignin blends showed potent possibilities as biomedical materials to prevent the cells against oxidative stress [128]. Original lignin was grafted with PLA directly using ring-opening polymerization. Controllable chain lengths of PLA in Lignin-g-PLA biocomposites were produced in the graft polymerization process, catalyzed by triazabicyclodecene. With the content of lignin increasing from 10% to 50%, glass transition temperatures of the grafted biocomposites increased from 45 to 85°C. An increase in the tensile strength (+16%) and strain (+9%) was shown after the existence of lignin copolymers without a sacrifice of the tensile modulus [129]. Softwood KL was modified successfully by the butyration reaction. PLA/butyration-lignin blends were made by melt mixing. The presence of broad but single *T*_g_ of PLA/B-lignin biocomposites was revealed by DSC analysis, indicating a highly compatible blend. The impact of PLA on the lignin thermal degradation was offered by TGA curves. Considering lignin content, storage modulus and overall high compatibility, PLA/B-lignin 25/75 biocomposites may be the optimal percentage to produce carbon fiber in the future [130].

PHB/modified lignin biocomposites are investigated by several reports. Thermal and mechanical tests described these biocomposites extensively. Acetylation modification using acetic anhydride was conducted on extracted rice husk lignin by the means of acidolytic method. Then the modified lignin was utilized to manufacture the PHB biocomposites with the solvent-casting method. The data found that thermo-oxidative degradation of the PHB was strongly interfered with by the acidolytic sample. Enhanced thermal stability was demonstrated, depending on the lignin amount in the bioplastics [131]. With the goal of improving the mechanical properties of PHB, Kai et al. (2018) developed a lignin-PHB copolymer. The lignin core was grafted with β-butyrolactone by ring-opening polymerization that was solvent-free. Nanofibers were produced by blending PHB with different amounts of lignin-PHB copolymers through electrospinning. Original PHB exhibited much higher mechanical properties than the lignin-PHB nanofibers. The greatest mechanical properties were demonstrated in nanofibers reinforced with 2% lignin copolymer. In addition, tunable antioxidant activity was indicated in the lignin/PHB nanofibers, which can be employed to neutralize extra free radicals in vivo. The non-irritating and biocompatible lignin/PHB nanofibers were also revealed by the animal experiments. As a result, the lignin/PHB nanofibers held possible applications in biomedicine [120]. A lignin-polycaprolactone copolymer was synthesized through the ring-opening polymerization without solvent. To improve their mechanical properties, these lignin-polycaprolactone copolymers as reinforcement fillers were combined with PHB nanofibers. It was shown that the lignin copolymer with PHB nanofibers revealed the optimal mechanical strength. Elongation at break of PHB nanofibers enhanced from 15% of 55%, and tensile strength increased from 1.81 to 3.13 MPa. Lignin acted a rigid core, polycaprolactone played as a rubbery layer in the complex system and fiber matrix formed strong hydrogen bonding with PHB nanofibers. In addition, lignin/PHB nanofibers with superior biocompatibility and biodegradability were demonstrated, displaying that the novel nanofibrous composites showed important applications in biomedicine [132].

Thermoplastic starch (TPS)/esterified lignin composites improve the mechanical properties compared to TPS. Esterified lignin was used as fillers in the TPS matrix. The TPS/lignin bioplastics were fabricated by compression molding; 5 wt % lignin content was used in the composites. The TPS/esterified lignin and TPS/KL bioplastics presented higher tensile strength than that of the TPS by about 32% and 17%, respectively. Moreover, water uptake properties decreased significantly owing to the existence of lignin in the TPS bioplastics [133]. A hydroxy-methylated alkali lignin/corn starch cross-linked film was produced. Results showed elongation at break and tensile strength of the corn starch film were lower than that of the composite film. In addition, thermal stability of the lignin/starch bioplastics at high temperatures was improved. The water uptake ability of lignin/starch bioplastics was 1.3 times of the TPS. Hence, the resulting lignin/starch biocomposites could be utilized as a biodegradable material for agricultural mulching and packaging [134]. Thermoplastic starch and layered double hydroxide obtained from lignosulfonates showed excellent dispersion in the nanoscale, with lignin concentrations between 1 and 4 wt %. Thus, the lignin was a promising bio-based reinforcement for TPS [135].

Lignin has already been used to produce polymers (e.g., polyurethanes and polyesters) typically by functionalization of the hydroxyl groups [4,32]. Moreover, lignin has been an alternative phenol for kinds of thermoset resins for a long time such as phenol-formaldehyde resins and epoxy resins due to its being the most important and renewable source of phenolic groups [32,53].

In general, modified lignin, which commonly involves complex reactions or toxic materials, shows better adhesion and enhanced properties in the bioplastics compared to pure lignin. However, its application in industry needs to consider the cost and benefit comprehensively because bioplastics demand low cost of production. More environmentally friendly methods and technologies of lignin modification are required urgently.

## 4. Application of Lignocellulosic Fibers in Bioplastics

The application of lignocellulosic fibers in bioplastics has been investigated in several studies which mainly focus on cellulose fiber. Several reviews published recently can provide the readers with more detailed information [11,136]. Unmodified fibers can serve as a cheaper material compared to cellulose modification, therefore, the application of raw lignocellulosic fibers in bioplastics has attained more attentions in recent years [11]. Although the studies on the application of lignocellulosic fibers in bioplastics have been conducted for decades, the interfacial compatibility between matrix and fiber is still not addressed [38,40,137]. First, either inter- or intra-molecular hydrogen bonding has been developed in most functional groups of LCC. These strong interactions in LCC render lignocellulosic fibers recalcitrance for interaction with other biopolymers. As a consequence, additional hydrogen bonding between these biopolymers is difficult to create via blending, which results in low interfacial miscibility, and further deteriorates the mechanical properties of the biocomposites. Second, due to the intense interaction among lignocellulosic fiber molecules, agglomeration is possible to exist in the biocomposite, resulting in low performance of bioplastics. As free movement of fibers will be severely restricted by the high viscosity of biopolymers, the case in the melt process is much more serious. Therefore, concentrated areas would be generated due to the agglomerated fibers in the biocomposites and they lower the performance of bioplastics further [11]. Third, due to the presence of hydroxyl and other chemical groups of LCC, lignocellulosic fibers exhibit high polar and hydrophilic characters. In addition, the amorphous components in the lignocellulosic fibers such as pectin, lignin, hemicellulose, and other waxy materials also allow absorption of moisture [3]. Swelling and holes in the LCC are likely to develop due to the water uptake of LCC, which induces limited dimensional stability and poor mechanical properties of the bioplastics [24,26].

**Figure 4:**
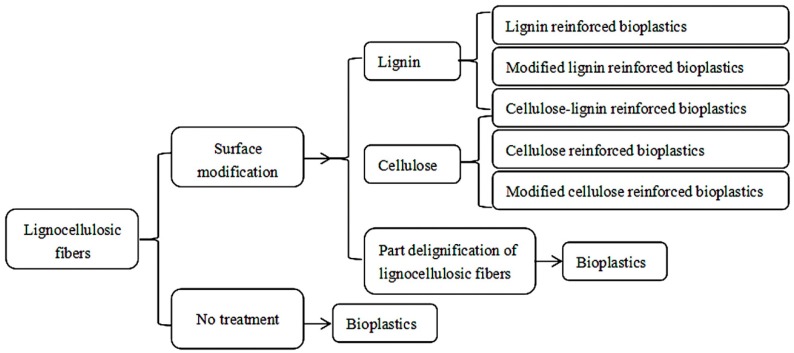
Global scheme of the uses of lignocellulosic fibers and lignin in bioplastics.

The addition level of lignocellulosic fibers in bioplastics is seriously restricted by the low compatibility. There are a variety of pretreatment methods to overcome these problems. However, most of the products or residues of these pretreatments have been based on biomass biorefining which removes lignin. This process is technically demanding and profitably impractical because lignin is also widely used in bioplastics [38,138]. Therefore, in this section we will review a few examples about lignin-containing cellulosic fibers to produce bioplastics (Figure 4). To some extent, we can regard them as lignocellulosic fibers which remove water soluble matter and hemicellulose. Therefore, these lignocellulosic fibers have a higher hydrophobic character and lower intermolecular interaction. In the end, we summarize the ways attempted to enhance the interaction between lignocellulosic fibers and biopolymers or to reduce the degree of association of lignocellulosic fibers without delignification when developing the bioplastics.

### 4.1. Lignin-Containing Cellulosic Fibers as Reinforcements of Bioplastics

Lignocellulosic residues containing a remarkable polysaccharide portion represent a very interesting source of fillers for bioplastics [84]. The interaction between lignin-containing cellulosic fibers and biopolymers are complex owing to the various composition of cellulosic fibers. The interaction between jute strands and PLA was evaluated. Five different lignin contents of strands were introduced into the PLA with the fiber ratio of 30 wt %. A PLA matrix was prepared in a discontinuous extruder and characterized by tensile tests. Jute strands with a lignin content of 4% were the most suitable to be used as PLA filler as shown by macro and micromechanical analysis. The phenomenon was mainly attributed to their better interaction and dispersion within the PLA matrix and higher intrinsic mechanical properties [138]. Lignocellulosic fillers obtained from bioethanol production were blended with PHB. Spectroscopic, thermal and morphological characterization showed there was a high polysaccharide content in the filler. Lignocellulosic filler produced an active effect on the PHB physical aging by acting as a heterogeneous nucleating agent. Infrared spectroscopic data showed that a low interaction between the lignocellulosic filler and PHB was observed. Furthermore, a deterioration of impact and tensile properties in the composites was attributed to this lack of connection. On the other hand, the biocomposites became more resistant to the degradation with concentration of lignocellulosic filler increasing, possibly because of the antibacterial activity of the lignin. Therefore, because lignocellulosic fillers improved the biopolymer properties in a cost-effective and environmentally friendly way, the utilization of these agro-industrial residues held great potential to widen the application of PHB [139].

Lignin-containing cellulosic nanofibrils (LCNF) can produce enhanced properties when incorporated into bioplastics. Residual oil palm empty fruit bunches were extracted to obtain lignin-containing cellulosic nanofibrils (LCNF) with a variety of seperation methods. Different kinds of LCNF isolated were incorporated into starch bioplastics. The Young’s modulus and yield stress achieved remarkable increases after the incorporation of LCNF. In addition, with LCNF loading increasing, water uptake of the composite bio-foams was reduced because of the low hydrophilicity of lignin residuals. The starch/LCNF nanocomposites displayed the same mechanical properties as those of polystyrene polymers. Therefore, they can be recognized as a potential and green alternative in insulation and packaging composites [84]. In order to develop composite films by casting and hot press, PLA and different amounts of LCNF from 5 to 20 wt % were blended. A good interfacial adhesion between PLA and LCNF was indicated due to the existence of lignin and also evidenced by results of infrared spectroscopy and atomic force microscope characterization at the nanoscale. Water resistance, thermal and mechanical properties were confirmed to improve remarkably in the resultant bioplastics at 5–10 wt % LCNF addition [34].

Though the reports on lignin-containing cellulose-reinforced bioplastics are scarce, they are adequate to indicate their promising application in bioplastics in future. We might conclude that they might significantly affect and even determine whether direct use of lignocellulosic fibers in bioplastics will be achieved. They should be the hotspot in the future. Lignin-containing cellulosic nanofibrils would play a significant role in preparing the bioplastics with lignocellulosic fibers.

### 4.2. Lignocellulosic Fibers without Delignification as Reinforcements of Bioplastics

During the preparation of bioplastics, physical interactions between lignocellulosic fibers and the biopolymers matrix are usually restricted and do not improve the performance of biocomposites significantly [122,140]. Therefore, the ways attempted to improve the compatibility are mainly focused on increasing the fiber–matrix interaction, reducing degree of association and improving the hydrophobic character of lignocellulosic fibers.

The interaction between matrix-fiber can be promoted with the compatibilizer or coupling agent interacting with both fiber and polymer [141]. González et al. (2011) reported the thermal and mechanical properties of fiber and PLA-reinforced biocomposites. PLA-based composites were manufactured by injection molding. Both sisal and kraft fibers composites revealed higher impact strengths than that of neat PLA (24.61 and 14.45 J/m, respectively). In addition, a good dispersion of reinforcements in the PLA composites was revealed by microscopic analysis [142]. Both flexural strength and modulus of starch biocomposites were significantly increased by laccase treatment with the mediators including violuric acid and fibers. The water uptake of biocomposites was reduced considerably. This biocomposites represented a promising means to produce non-food and disposable packaging materials based on above biopolymers [143]. The effect of coupling agent and fibers on water uptake behavior was assessed in polyhdroxybutyrate-*co*-polyhydroxyvalerate, or PHBV/sisal biocomposites, by analyzing the water absorption capability and water diffusion rate. It was found that more water would be incorporated when the biocomposites absorbed the water more quickly; as the content of sisal fibers increased in the biocomposites, the sample temperature became higher. In particular, saturated water absorption was more associated with the ratio of fibers, while temperature was primarily determined by water diffusion. Owing to chemical bonding between fiber and matrix, the interaction was affected by diffusion rate, but saturated water incorporation did not show significant impact on the interaction [140].

The effects of fiber properties such as fiber length or aspect ratio on increasing fiber–matrix interaction are also investigated. Fiber length is an important parameter to enhance toughness and strength, while fiber dispersion mainly promotes strengths [144,145,146]. Soy and kenaf fiber bioplastics were manufactured by compression or injection molding. Mechanical properties and dynamic mechanical analysis characterized the effect of the processing method and fiber length on the performance of the biocomposites. The modulus and impact strength of the bioplastics rose with fiber orientation, fiber content and fiber length increasing. Microscopy observations revealed that fiber length could positively influenced the fracture surface as fiber content and fiber length increased. This suggested the predominant fiber bridging effects on impact strength of the bioplastics [95]. A complex biocomposite by a compounding and molding process was investigated with the aim of achieving high impact strength. Low and high screw extrusion methods were involved. Fiber with 2.3 mm fiber length and 20 wt % fiber content indicated a good dispersion in the biocomposite whose impact strength was 130 J/m. It can be concluded that toughness was influenced by fiber dispersion, length and concentration [145]. In order to investigate the effect of fiber amount and length, curaua fibers and poly(butylene succinate) were used to develop the biocomposites by compression molding. Mechanical strength, morphology and water uptake studies were performed to evaluate the characteristics of the biocomposites. It was found that the flexural and impact strengths increased as the fiber content increased. In addition, in the biocomposites with fiber content of 20 wt %, its impact strength was also affected by the fiber length ranging from 1 to 4 cm. However, the length of the fibers did not affect flexural strength significantly. Water uptake analysis confirmed the material was more sensitive to fiber content compared to fiber size. The resulting bioplastics could be applied to interior car parts or rigid packaging because they possessed the same mechanical properties as poly(butylene succinate) [147].

Regarding lignocellulosic fiber reinforced bioplastics, it is typically acknowledged that stress transfer from the matrix to the fiber is enhanced by fibers having a high aspect ratio (length/width). A high aspect ratio is very crucial in lignocellulosic fiber reinforced biocomposites because it indicates possible strength performance [3]. Biocomposites were prepared using twin extrusion process with lignocellulosic hemp fiber as reinforcements and poly(ε-caprolactone) as matrix. Different aspect ratios (19–38) of fibers were prepared. Properties including low-velocity impact, flexural and tensile were enhanced in the poly(ε-caprolactone)/hemp fiber biocomposites. The best properties were revealed in the biocomposite with 26 aspect ratios, with modulus and flexural strength of 285% and 169%, respectively. However, mechanical properties presented a significant decrease in water immersed biocomposites; flexural moduli and tensile reduced by 62% and 90%, respectively. The results provided a green replacement of a petroleum-based and conventional polymer matrix in potential applications [148]. In addition, the adhesion between fibers and polymers and the dispersion of fiber in matrix are also vital to an effective stress transfer. The intense shearing forces can reduce the aspect ratio of cellulosic fibers in the mechanochemical process. The milling process can cause the degradation of matter with lower molecular weight, which produces low mechanical properties. However, the negative effect from milling can be compensated by the crosslinked structure and effective fiber dispersion, providing the bioplastics with enhanced performance [149].

In addition, modification of lignocellulosic fibers is studied to improve hydrophobicity and compatibility of the lignocellulosic fiber-matrix with thermal treatment or ultrasonic. To improve the properties and fiber/matrix adhesion of the bioplastics, wheat straw fibers/PHBV bioplastics were fabricated. Wheat straw fibers were treated by torrefaction treatment to increase their hydrophobicity. SEM observations revealed that an excellent fiber/matrix compatibility was achieved in the resulting biocomposites. The crystallinity and molecular weight of the bioplastics remained constant and the crystallization of PHBV was facilitated by the introduction of torrefied fibers compared to untreated fibers. The mechanical properties of wheat straw fiber/PHBV biocomposites were only affected by the 30 wt % of torrefied fibers, which displayed increased rigidity. A 30% decrease in water vapor permeability was observed with 20 wt % torrefied fiber contents attributed to the hydrophobic nature of fibers and improvement of fiber/matrix adhesion [150]. The chemical composition of the fibers does not change significantly in physical treatments. Therefore, as mechanical bonding between the matrix and the fiber increased, the adhesion between them is improved [3]. Water hyacinth fiber with 10% volume fraction was employed to reinforce the biocomposites obtained from tapioca starch-based plastics. During gelatinization, the biocomposites were cast into a glass plate then placed in an ultrasonic bath. After vibrated with ultrasound for 30 min, the biocomposites with optimal properties can be produced. Tensile modulus and tensile strength increased by 108% and 83% after this vibration. At the same time, water resistance of the bioplastics increased by 25% and achieved a maximum [151]. The empty fruit bunch fibers and tapioca starch were blended to prepare the bioplastics. After the gelatinization of the biocomposites, the solution was cast into a glass plate and then vibrated in an ultrasonic bath with 250 watts and 40 kHz in different duration for 0, 15, 30, 60 min, respectively. The results demonstrated the characterization of the biocomposites has been influenced by the vibration during gelatinization. The different fracture surface of the tensile sample was revealed by SEM studies. For vibration duration of 60 min, strain was decreased to 35.1%, meanwhile tensile modulus and tensile strength increased to 277.4 and 64.4%, respectively, compared to untreated one. Water uptake of the untreated biocomposites was higher than that of the vibrated one. FT-IR and XRD of the biocomposites have changed due to various vibration duration [152].

The number of researches on lignocellulosic fibers and lignin reinforced bioplastics are much fewer compared to cellulose reinforced bioplastics. The difference can be explained by the complexity and difficulty to fully utilize lignocellulosic fibers and lignin. The development of processing technologies and innovative ways to enhance the properties of lignocellulosic fibers and lignin would undoubtedly further boost their potential applications in bioplastics [11,60].

### 4.3. Comparison between Lignocellulosic Fibers and Lignin in Bioplastics

Based on above discussion, lignocellulosic fibers and lignin have showed great success in making the bioplastics. In this section, we will make a comparison on structure, role, modification and future of lignocellulosic fibers and lignin in the bioplastics. Similarities are examined due to their structure and addition amount. Firstly, there are strong inter-molecular interactions in lignocellulosic fibers and lignin which render recalcitrance for interaction with other biopolymers. This usually reduces the performance of the composites. Agglomeration occurs in the biocomposites due to the intense interaction within lignocellulosic fiber or lignin molecules. Free movement of fibers will be restricted by the high viscosity of biopolymers during the melt process [24,26]. Secondly, the addition of lignocellulosic fibers and lignin in the bioplastics can be as high as 10% or above with improved properties in some reports, but additional contents of nanoscale particles are restricted below 3% due to the presence of aggregation. As we know, most of lignocellulosic fibers and lignin have low added-value [53]. If they are used as fillers or alternatives for biopolymers, the microparticles have a more promising future in bioplastics.

Differences between lignocellulosic fiber and lignin-reinforced bioplastics are identified as follows. Firstly, lignocellulosic fibers and lignin play different roles when introduced into other biopolymers. Lignin can function as reinforcement, plasticizer, stabilizer, coating or bio-compatibilizer, which widens its application. By contrast, lignocellulosic fibers primarily act as reinforcement or replacement for the biopolymers in the bioplastics, which is ascribed to the more complex compositions and recalcitrant structures of lignocellulosic fibers compared to lignin [74]. Secondly, chemical modifications of lignin such as functionalization of hydroxyl group are more widely studied than those of lignocellulosic fibers, which is also related to their composition and structures. The interaction between lignin and biopolymers is hydrogen bond, which can be promoted by compatibilizers, crosslinkers or chemical modification. However, the interaction of lignocellulosic fibers and biopolymers is not addressed clearly. Compatibilizers and crosslinkers are used to improve the interaction of lignocellulosic fibers and biopolymers. Physical treatments such as thermal and ultrasonic treatment also reduce the degree of association of lignocellulosic fibers. In the end, the hydrophilic nature of lignocellulosic fibers is also a great challenge needed to overcome in the bioplastics. Conversely, the lignin is hydrophobic [60]. As a consequence, lignin presents more advantages than lignocellulosic fibers in the bioplastics. It seems to be unwise to remove lignin when the researchers fabricate the bioplastics with lignocellulosic fibers. One way is to separate lignin by destroying the degree of association of lignin and other components and mix lignin with the residuals to produce the bioplastics. Another way is to apply the lignocellulosic fibers directly or with simple modification in the bioplastics.

## 5. Conclusions

In this review, the structures and properties of lignocellulosic fibers and lignin are characterized. Their recent advances and issues in making bioplastics are elaborated comprehensively. In conclusion, the lignin as reinforcements can produce bioplastics with high performance. Lignin has the possibility to function as a plasticizer, stabilizer, or bio-compatibilizer in bioplastics, which will produce different properties on bioplastics. In addition, lignin is modified to enhance its miscibility with biopolymers considerably by functionalization of hydroxyl groups. However, due to its complex structure, the ability to obtain technical lignin of a reduced degree of association is presently a very tough challenge for promoting the use of lignin in bioplastics. Although studies on the application of lignocellulosic fibers in bioplastics have been conducted extensively for many years, the low fiber-matrix compatibility is still not addressed. Recent research on cellulose-lignin and lignin-containing cellulosic fiber-reinforced bioplastics are very attractive and meaningful. Fiber–matrix interactions by compatibilizer, crosslinking and proper fiber length are overcoming the low compatibility further. Simple modifications of lignocellulosic fibers, which do not extensively change the chemical content or composition of the fibers, are very promising for making more functional groups of fibers exposed for interaction with biopolymers.

We can conclude that more studies should be directed at lignocellulosic fibers without removing lignin when preparing the bioplastics. However, the application of lignin in the bioplastics still has a prosperous future because of its various sources from paper-making and ethanol production. Due to some similar application issues in the bioplastics, such as complex structure, lignocellulosic fibers and lignin will promote and develop mutually in the future.

## Figures and Tables

**Table 1:** Chemical composition of some lignocellulosic fibers [38,39,40,41].

Fiber	Cellulose (wt %)	Hemicellulose (wt %)	Lignin (wt %)
Bagasse	55.2	16.8	25.3
Bamboo	26–43	30.0	21.0–31.0
Birch branches	33.3	23.4	20.8
Corn stalk	42.7	23.6	17.5
Flax	71.0	18.6–20.6	2.2
Kenaf	72.0	20.3	9.0
Hemp	68.0	15.0	10.0
Jute	41–48.0	21–24	18.0–22.0
Oil palm	65.0	-	29.0
Pine branches	32	32	21.5
Rice rusk	35.0–45.0	19.0–25.0	20.0
Rice straw	41.0–57.0	33.0	8.0–19.0
Sisal	65.0	12.0	9.9
Spruce branches	29	30	22.8
Switchgrass	34.0	27.0	17.0
Wheat straw	38.0–45.0	15.0–31.0	12.0–20.0

**Table 2:** Glass transition temperature and delignification process of different lignins [53,60,61].

Type of Lignin	Glass Transition Temperature (°C)	Delignification Process
Hardwood Kraft lignin	108	NaOH, Na_2_S
Softwood Kraft lignin	153	NaOH, Na_2_S
Hardwood Lignosulfonates	138	HSO_3_^−^, H^+^
Softwood Lignosulfonates	127	HSO_3_^−^, H^+^
Wheat straw Soda lignin	150	NaOH
Hardwood Organosolv lignin	95	Organic solvent, water
